# Agent-Based Modeling in Public Health: Current Applications and Future Directions

**DOI:** 10.1146/annurev-publhealth-040617-014317

**Published:** 2018-01-12

**Authors:** Melissa Tracy, Magdalena Cerdá, Katherine M. Keyes

**Affiliations:** 1 Department of Epidemiology and Biostatistics, School of Public Health, University at Albany, State University of New York, Rensselaer, New York 12144, USA; 2 Department of Emergency Medicine, University of California, Davis, Sacramento, California 95616, USA; 3 Department of Epidemiology, Mailman School of Public Health, Columbia University, New York, NY 10032, USA

**Keywords:** complex systems, computer models, epidemiology, population health, simulation, systems science

## Abstract

Agent-based modeling is a computational approach in which agents with a specified set of characteristics interact with each other and with their environment according to predefined rules. We review key areas in public health where agent-based modeling has been adopted, including both communicable and noncommunicable disease, health behaviors, and social epidemiology. We also describe the main strengths and limitations of this approach for questions with public health relevance. Finally, we describe both methodologic and substantive future directions that we believe will enhance the value of agent-based modeling for public health. In particular, advances in model validation, comparisons with other causal modeling procedures, and the expansion of the models to consider comorbidity and joint influences more systematically will improve the utility of this approach to inform public health research, practice, and policy.

## INTRODUCTION

Agent-based modeling is an increasingly popular method for visualizing, analyzing, and informing complex dynamic systems in public health. Although agent-based models (ABMs) hold promise for providing insight into population-level health outcomes and interventions, careful consideration of the limitations and challenges of these models is required to realize their full potential. In this article, we provide an overview of: agent-based modeling in public health, including the central properties and assumptions of the models and how they complement other complex systems approaches; key areas in public health where this method has been adopted; the advantages of this method for questions with public health relevance; the limitations and challenges of these models; and critical future directions, both methodologic and substantive, that we believe will enhance the value of ABMs for public health research, practice, and policy. For additional reading, we direct the reader to commentaries (1, 24, 25, 27, 31, 41) and systematic reviews of agent-based modeling in public health (84, 95) as well as tutorials on agent-based modeling (42, 72, 88).

## PROPERTIES OF ABMs

**Figure 1:**
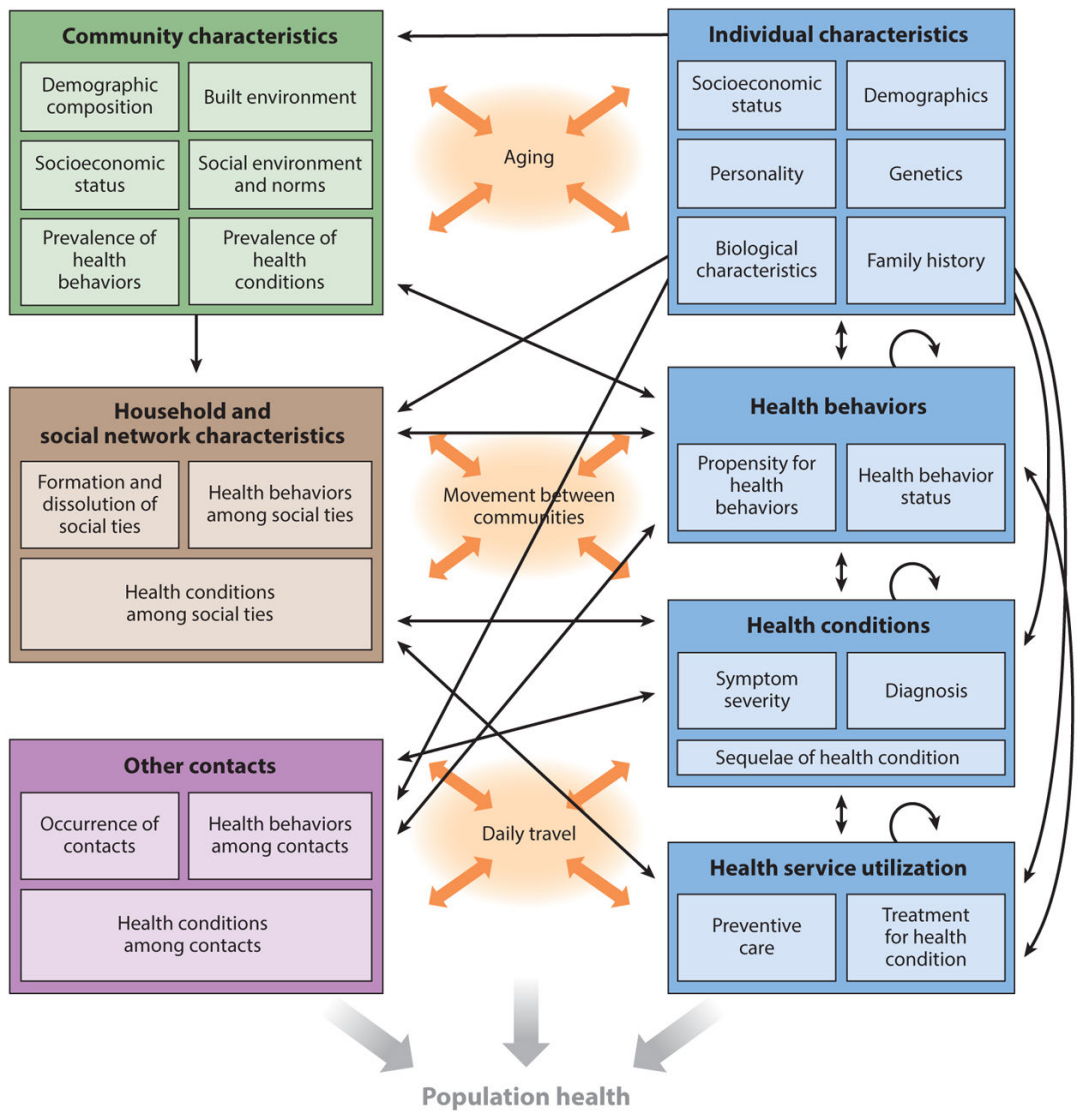
An illustration of a hypothetical agent-based model. Individual characteristics such as demographics, health behaviors, health conditions, and health service utilization (*blue*) influence and are influenced by community characteristics ( *green*), social ties (*brown*), and other contacts ( *purple*), as well as ongoing processes such as aging and movement through the environment (*orange*). Taken together, these static and time-varying characteristics at multiple levels and the often bidirectional processes that connect them create a system from which population health emerges.

Agent-based modeling is a computational approach in which agents with a specified set of characteristics interact with each other and with their environment according to predefined rules (1, 27, 67). Agents may represent individuals, households, governments, or any other entities of interest. They may adapt their behavior in response to their experiences, interactions with other agents, and interactions with their environment. A defining feature of agent-based modeling is that it allows the emergence of population-level phenomena that are greater than or different from what would be expected based only on the aggregate of individual behaviors (7). Agent-based modeling is thus referred to as a bottom-up approach, in which behaviors at the micro level give rise to dynamics at the macro level (32). As illustrated in Figure 1, which presents a diagram of a hypothetical ABM of individuals embedded in multiple contexts, ABMs may include a variety of individual-level characteristics (ranging from endogenous factors to socioeconomic status) as well as community-level characteristics and other social influences that work together to shape individual health behaviors, health outcomes, and health service utilization. ABMs can also explicitly incorporate the effects of ongoing processes like aging and movement between communities. Together, these interactions and biologic, behavioral, and social processes make up the system from which population health emerges.

Other distinct properties of ABMs include autonomy, heterogeneity, feedback, and stochasticity. Autonomy implies that agents make decisions about how to act given their current circumstances and programmed behavioral rules (72, 84). Heterogeneity is reflected in the differences among agents and among parts of the environment, which may have multiple static and time-varying characteristics in an ABM (1, 27). Changes in agent and environmental characteristics may be amplified in unexpected ways over time through feedback, whereby past experiences change the course of future responses (2, 24). Stochasticity allows the model to unfold in a probabilistic (as opposed to deterministic) fashion, with randomness influencing behaviors and changes in the model (1). As a result of these properties, ABMs can be used to consider nonlinear relations influenced by multiple levels and interpersonal interactions in ways that are often more flexible than those offered by other approaches. As such, ABMs (and complex systems approaches more generally) permit a broader array of research questions than traditional analytic approaches can answer, with the potential to shed new light on population health problems.

Agent-based modeling shares objectives and capabilities with other complex systems approaches, including system dynamics modeling and network analysis. System dynamics models use a series of differential equations to reflect stock variables (e.g., population subgroups) and flows into and out of stocks, including bidirectional relationships (54). Such models are particularly well suited to modeling high-level system behavior in large populations (66, 67). However, typically system dynamics models do not finely specify the micro-level behaviors of individuals, including interactions between individuals and adaptations over time. By contrast, network models can accommodate complex network structures, including the transfer of information, behaviors, and disease across connections (27, 67). Network analysis can be used to examine how and why networks change over time and to test hypotheses regarding their structural and social influences on the development of health behaviors and outcomes. However, network analysis is not well suited to considering higher-level system properties. ABMs complement and extend these approaches by incorporating network dynamics while also accounting for multiscale interactions and bidirectional feedback loops.

Systems science approaches like ABM have long been used in fields outside of public health. ABM grew out of the computational and information processing advancements in computer science, mathematics, physics, game theory, and evolutionary science that occurred during the twentieth century, including Von Neumann’s cellular automata, Conway’s Game of Life, and Holland’s genetic algorithms (67, 79). As evidence accumulated that population health outcomes reflect more than the sum of individual risks, emerging from interactions between individuals over time (56), ABMs began to be viewed as a useful tool for public health problems as well. Applications of ABMs in ecology, business, political science, and the social sciences (7, 8, 30, 69, 73, 88) have been influential in the development of ABMs in public health, as well as in the formalization and standardization of these approaches across disciplines (46–48).

## APPLICATIONS OF ABMs IN PUBLIC HEALTH

In public health, agent-based modeling has historically been used almost exclusively to model infectious disease transmission and control in populations. ABMs are a natural fit for modeling infectious disease transmission because interactions between individuals, and individual interactions with local environments, often give rise to population patterns of infectious disease incidence and persistence. However, in the last 15 years, these methods have been increasingly applied to noncommunicable diseases, health behaviors, social epidemiology, and other issues relevant for population health that do not involve traditional infectious processes (84). These ABMs fall along a wide continuum, from abstract representations of a simplified system (43) to realistic simulations of a well-defined population (6).

### ABM Applications in Infectious Disease Epidemiology

Many ABMs of infectious disease transmission rely on the susceptible-infected-recovered (SIR) framework proposed by Kermack and McKendrick in the 1920s (94), in which the flows between susceptible, infected, and recovered states are governed by differential equations (32). ABM extensions of SIR models have been used to introduce individual heterogeneity and more complex network interactions into these traditionally aggregate, compartmental models, providing further insight into infectious processes in real-world settings (15, 32). ABMs of infectious disease have also been widely used to evaluate infection control policies and have thus informed the development of containment strategies by the Centers for Disease Control and Prevention (CDC) and other government agencies (87). Notable ABMs in infectious disease epidemiology include comparisons of vaccination strategies to address a deliberate bioterrorist introduction of smallpox (33, 51), tuberculosis control strategies (82), use of targeted antiviral prophylaxis and social distancing measures to prevent an H5N1 influenza A (bird flu) pandemic (38), contact tracing and quarantine to reduce measles transmission (28), treatment and hygiene procedures to reduce *Clostridium difficile* infection transmission in health care settings (17), evacuation plans in the event of airborne contamination (34), and vaccination strategies against influenza pandemics, including their impact on health care personnel (19, 59). This work includes the Models of Infectious Disease Agent Study (MIDAS), which has brought together a collaborative network of researchers to inform national responses to outbreaks of existing and emerging infectious diseases (101). Recent ABMs have also considered interventions to reduce human immunodeficiency virus (HIV) incidence (35, 74, 76), including combination strategies addressing both HIV transmission risks and underlying drug use behaviors. ABMs of infectious disease have thus advanced to include increasingly sophisticated parameterization of social networks and environmental influences to best inform public health policy and planning. Furthermore, many of the modeling capabilities developed, extended, and refined through infection-related ABM programs like MIDAS [e.g., census-based synthetic populations, and the Framework for Reconstructing Epidemic Dynamics (FRED)] (45, 104) can be applied to public health problems beyond infectious disease.

### ABM Applications in Noncommunicable Disease Control

The increasing recognition that dependence between individuals and feedback over time are also important to noncommunicable diseases (16, 83) has led to increased applications of ABMs in this area. Obesity and its correlates have been the subject of a plurality of these investigations (84), given the urgency of obesity as a public health problem and the complex influences of biological, behavioral, social, and environmental factors on the risk of obesity over the life course (44, 67). Some of this work was developed by the National Collaborative on Childhood Obesity Research through the Envision project, which aimed to apply systems science methods to identify potential points of intervention to reduce population levels of obesity (70). ABMs of obesity have emphasized the importance of accounting for the clustering of obesity in social networks and neighborhoods, including simulations of social network influences on body weight (52) and joint neighborhood and individual influences on Black/White disparities in body mass index (86). ABMs have also been utilized to study diabetes, illuminating the progression of diabetic retinopathy and the influence of screening on vision loss among diabetic patients (22, 23), as well as the influence of patient-provider interactions on the adoption of continuous glucose monitoring (102). These models have thus made inroads into the understanding of noncommunicable disease development, progression, and treatment, including the role of communities, peers, and providers.

### ABM Applications to Health Behaviors

In addition to assessing disease endpoints in the population, ABMs have also been used to gain insight into health behaviors that increase the risk of disease, as well as potential interventions to reduce risky behaviors like smoking, alcohol consumption, physical inactivity, and unhealthy eating.

ABMs have highlighted the role of social influences on tobacco control policies and smoking behaviors in the population. ABMs of smoking have explored the effects of transitions to electronic cigarette use on population smoking prevalence (14) and the roles of socioeconomic status and social influence on smoking behaviors (13, 90). By explicitly incorporating interactions between individuals, this work has extended conclusions from previous system dynamics models of smoking (61, 62). A recent Institute of Medicine report summarized the usefulness of ABMs for studying the complex, dynamic influences on smoking initiation, cessation, and relapse, and it included recommendations for developing and evaluating ABMs for tobacco control (18). One advantage of these methods over more traditional analytic approaches is their ability to identify the potential unintended consequences of alternative tobacco control strategies. This is important given the tobacco industry’s history of successfully adapting its marketing and lobbying plans to address control policies like increased taxation and restrictions on advertising, thereby undermining efforts to reduce smoking in the population (100).

ABMs have also considered the unintended consequences of policies aimed at alcohol consumption and related harms. The SimDrink ABM simulated the behaviors of young people (aged 18–25 years) during a night out, including the types of venues (e.g., private versus public) visited by groups of friends and the decisions they made about when to go home (93). Population experiences of alcohol-related harms (e.g., verbal aggression and ejection from outlets because of intoxication) were compared under different simulated policies, including extending public transportation hours and instituting “lockout” times after which no one could be admitted to a venue (e.g., two hours before closing). The authors explicitly addressed the possibility that these policies may increase alcohol-related harms by keeping drinkers out later or may merely displace harms from public to private settings. Gorman and colleagues used a more abstract ABM to explore social and environmental influences on drinking behavior (43). Agents could move left or right on a one-dimensional lattice, with movements and transitions between drinking states (e.g., nondrinker, current drinker, former drinker) influenced by the drinking behaviors of other agents they encountered on the lattice. The authors also introduced an on-premise alcohol outlet (i.e., a bar) onto the lattice that attracted current drinkers. ABM results indicated that contact between nondrinkers and drinkers would eventually eliminate nondrinkers from the population, though the amount of time required for that to occur varied according to the frequency of agent movement and contact between agents and to whether there was a bar on the lattice around which current drinkers clustered, thus limiting their interaction with nondrinkers and former drinkers. These findings highlight how contacts between individuals and between individuals and alcohol outlets shape population levels of drinking behavior. This supports theoretical work by Gruenewald (49) on “assortative drinking,” whereby individuals with similar preferences and behaviors cluster together at particular drinking venues, reinforcing potentially hazardous drinking norms.

ABMs have been used to demonstrate the importance of environmental influences on physical activity, independent of individual and peer preferences. For example, Yang and colleagues (107) developed an ABM that modeled individual walking behaviors in a simulated city. Walking ability was a function of age, whereas walking preferences were influenced by previous walking experiences, by seeing others walking, and by the walking attitudes of friends and family members, thereby incorporating learning and adaptation. Population patterns, including inequalities in walking across socioeconomic status, were observed under different distributions of land use and safety, thereby accounting for environmental influences. This model was extended to also consider the influence of different interventions (e.g., improving the safety level of certain areas, increasing positive attitudes towards walking) on walking behaviors (108). Yang and colleagues also developed other ABMs to investigate influences on children’s active travel to school (106), including optimizing the so-called walking school bus, in which students walk to school in groups led by adults, following a planned route with designated “bus stops” (109). These studies all highlighted the importance of land-use distributions and equity of environmental resources on walking behaviors and on the effectiveness of interventions aimed at increasing physical activity. Other scholars have developed additional ABMs to examine changes in transportation infrastructure on walking behaviors (60) and to implement a customizable tool aimed at enhancing walkability around designated areas (5), further capitalizing on the ability of these models to incorporate feedback between individuals and their environment.

ABMs of diet have also highlighted the importance of feedback between individuals and the environment. Auchincloss and colleagues used an ABM to explore determinants of income inequalities in diet (3). Households could decide to patronize specific stores based on food prices, store distance, and preferences for healthy foods, whereas stores could decide to relocate or change their offerings based on customer preferences. Model results indicated that income differentials in diet emerged as a result of the segregation of healthy food sources and high-income households from less healthy food sources and low-income households. The authors proceeded to show that changing food preferences among low-income households in combination with reducing the prices of healthy foods could eliminate income differentials in diet. Blok and colleagues developed a related model simulating household food consumption and food outlet distribution and changes in a city in the Netherlands (6). Income inequalities in healthy food consumption were reduced by eliminating residential segregation, lowering the prices of healthy foods, and increasing preferences for healthy food consumption through mass media education campaigns. Li and colleagues (63) also explored the potential effect of education campaigns on healthy food consumption by using an ABM to simulate individuals, social networks, and food outlets in New York City (NYC) neighborhoods. Individuals’ daily dietary choices were influenced by demographic characteristics, food access, price sensitivity, taste preferences, and health beliefs, with taste preferences and health beliefs in turn influenced by the individuals’ friends in the model. A mass media marketing campaign and community nutrition education program was assumed to increase the influence of healthy peers on food consumption choices by 10%, resulting in a substantial increase in fruit and vegetable consumption and thus highlighting the positive influence of social norms.

### ABM Applications in Social Epidemiology

As demonstrated by the ABMs exploring social network and place effects on health, agent-based modeling is particularly well suited to examining questions of interest in social epidemiology, which often involve collective behaviors, distributions of resources, and other social conditions that serve as fundamental causes of disease (39, 65). In line with this work, a recent ABM by our group has explored the social production and propagation of violence and tested alternate strategies for reducing violence and its consequences (10–12). Specifically, we created a virtual representation of the adult population of NYC, distributed across NYC neighborhoods. Violent experiences among individuals in the model were governed by interactions with other agents, sociodemographic characteristics, mental health symptoms, past histories of violence, and neighborhood characteristics, in addition to the actions of police officers and, in some model specifications, “violence interrupters” (i.e., community members trained to mitigate occurrences of violence and retaliation). The model compared universal and targeted experiments increasing neighborhood collective efficacy to reduce violence. The universal experiment was characterized by a small increase in collective efficacy across the whole city, whereas the targeted experiment intensified efforts to increase collective efficacy but only in high-violence neighborhoods (10). The results of these experimental conditions were contrasted under different hypothetical scenarios, including complete racial and economic residential segregation versus complete random mixing. We found that the universal experiment led to greater reductions in violent victimization across all groups, but racial/ethnic inequalities in violence persisted in the presence of racial and economic segregation. Only by reducing segregation through artificial random mixing across the environment were racial/ethnic inequalities in violence eliminated through increased neighborhood collective efficacy. In a subsequent iteration of the model, we investigated whether a population-level violence prevention intervention (i.e., hot-spots policing) versus an individual-level treatment intervention (i.e., increased access to cognitive behavioral therapy) could lead to a greater reduction in violence-related posttraumatic stress disorder (PTSD) in the population (12). Each approach resulted in only a modest reduction in violent victimization and violence-related PTSD, with the joint implementation of both approaches resulting in similar reductions in a shorter time frame. We then compared hot-spots policing with Cure Violence (9, 11), a community-based approach to violence prevention in which violence interrupters and outreach workers engage with high-risk individuals in the community to reduce the risk of violence. We found that combining the criminal justice approach of hot-spots policing with the public health approach of Cure Violence produced greater reductions in population levels of violence than either approach alone, reiterating the advantages of investing in multiple synergistic strategies.

Yonas and colleagues (110) also explored universal versus targeted approaches to crime in an ABM in which juvenile agents committed offenses according to their individually perceived risk and reward of doing so, which was influenced by the levels of adult criminal reporting near their location. Community-wide and spatially focused interventions aimed at increasing reporting by adults reduced offenses, with community-wide interventions affording greater reductions but also requiring greater resources. Lum and colleagues extended this work on the individual interactions that give rise to violence and other criminal offenses in an ABM that investigated the transmission of incarceration (68). The authors used a susceptible-infected-susceptible (SIS) model, in which individuals move between susceptible and infected states, to simulate incarceration transmission in a social network consisting of friends and family across multiple generations. The model reproduced observed racial inequalities in incarceration by applying differential Black and White sentences for drug possession, but it also showed that the transmission of incarceration between connected individuals was necessary to produce the incarceration and recidivism patterns observed in reality. The authors concluded that harsher sentences may increase, rather than deter, criminal behavior within social networks, and they recommended efforts to reduce the transmission of incarceration within networks, echoing other recent calls for further work aimed at understanding the transmission of violence within social networks (98).

These examples illustrate the wide applicability of agent-based modeling to public health problems, from infectious disease to violence. Scholars have also developed ABMs to inform the provision of health care services, including access to primary care services after a disaster (50), care coordination among patients with serious mental health problems (55), and participation in community-based oral health programs (78). Together, this body of work illustrates the ability of agent-based modeling to test competing theories and evaluate interventions in the presence of complex nonlinear influences.

## STRENGTHS OF ABMs APPLIED TO PUBLIC HEALTH PROBLEMS

Fundamentally, the two primary objectives of ABMs in public health are to explain and to predict population health outcomes, accounting for aspects of the complex system in which population health arises. These objectives also lead to the primary strengths of ABMs for public health research, practice, and policy: These models provide insight into the underlying mechanisms that give rise to health behaviors and outcomes (as well as inequalities in those behaviors and outcomes), and they can be used to conduct virtual experiments of interventions and policies to reduce the population burden of disease.

### Insight into Causal Mechanisms

Given their bottom-up nature, ABMs have been touted as one way to gain further insight into the mechanisms through which population patterns arise. In the words of Joshua Epstein (29, p. 43), “if you didn’t grow it, you didn’t explain its emergence,” which highlights the importance of generating a pattern to understand how that pattern came about (32). In a now classic example of using an ABM to generate an observed pattern, Schelling (91, 92) used a simple checkerboard model in which households preferred that a certain proportion of their neighbors be their own color to demonstrate the generation of population patterns of racial segregation that were much starker than individual preferences seemed to imply. Other ABMs mentioned above also exemplify this generation approach: Racial disparities in violence and income disparities in diet resulted from residential segregation in the ABMs developed by Cerdá and colleagues (10) and Auchincloss and colleagues (3), respectively, and patterns of incarceration and weight change were driven by social network influences in the ABMs developed by Lum and colleagues (68) and Hammond & Ornstein (52), respectively. In an example from infectious disease epidemiology, Kumar and colleagues (57) used an ABM to test whether differential exposure to influenza in areas with larger household size, higher population density, and younger age distributions was sufficient to generate area-level inequalities in influenza rates, finding that differential susceptibility was also necessary. ABMs are well suited to the exploration of causal mechanisms given their ability to incorporate multiple interacting causes and to test competing theories about causation, thus further elucidating what we do and do not know about how a given outcome arises (41, 83, 99). One challenge to the use of ABMs for identifying causal mechanisms is the fact that several model configurations may successfully generate the expected population patterns, so scholars cannot always be certain that they have hit on the right explanation (1, 27). However, with appropriate attention to the plausibility of model assumptions, ABMs have great potential for providing causal insights that are intractable using other approaches.

### Insight into Public Health Policy

In addition to providing clues into causal mechanisms, ABMs can be used to implement counterfactual simulations that may be infeasible in the real world, allowing what-if scenarios and virtual policy experiments. In particular, multiple simulations of the model can be run, observing population outcomes under different treatment conditions and thereby enabling counterfactual contrasts in which all other aspects of the population remain the same (27, 75). Not only can multiple policies or interventions be compared, but ABMs can also be used to identify the minimum “dose” of an intervention or the optimal combination of interventions needed to achieve a desired effect. For example, in Cerdá and colleagues’ (11) ABM of violence, a combined intervention approach led to an 11% reduction in the annual prevalence of violent victimization. Implementing each intervention alone would have required far greater resources and time to achieve the same result. Because ABMs are simplified versions of reality, researchers, policy makers, and other stakeholders are cautioned to interpret results of these in silico experiments qualitatively, as indicating what approaches may be maximally effective, rather than quantitatively, as providing precise numbers of health crises that will be averted or lives that will be saved (25, 27, 97). Recent methodological work in this area has attempted to explicate the conditions under which ABMs may best be interpreted as counterfactual simulations, including when multiple causal effects interact, when interference (i.e., the situation in which one individual’s exposure affects other individuals’ outcomes) is of explicit interest, and when system behavior is well defined and not overly sensitive to initial conditions (75).

Besides facilitating the comparison of population health outcomes under alternate intervention or policy scenarios, ABMs also allow exploration of the conditions under which these interventions may achieve their best results as well as their unintended consequences. As described above, we showed that racial disparities in violence remained intractable when residential racial and economic segregation was high, despite substantial increases in neighborhood collective efficacy under simulated interventions (10). Similarly, in Yang and colleagues’ (108) model of walking behavior, changes in attitudes towards walking or improvements in safety levels were not sufficient to induce changes in walking behaviors if other features of the environment (e.g., land-use mix) were not conducive to walking. The ability of ABMs to incorporate feedback and adaptation is particularly important in evaluating hypothetical interventions, especially when interventions may change behaviors, social networks, or environments in ways that may negate the desired positive intervention effects (96). ABMs can also provide insight into the net effect of an intervention that may positively influence one outcome (e.g., increased use of active transport modalities to reduce obesity) but negatively influence another outcome (e.g., increased risk of injuries through active transport) (77, 97).

The process of developing an ABM is often presented as a major strength of this approach (1, 2, 25, 26). Creating a conceptual framework for an ABM (like that pictured in Figure 1) brings together diverse stakeholders and lays bare assumptions about the aspects of a particular system and how they work together to produce population health outcomes. Model development and calibration highlight gaps in knowledge and empiric data about the underlying system. As a whole, this process generates new hypotheses and allows more expansive research questions to be considered.

## LIMITATIONS OF ABMs APPLIED TO PUBLIC HEALTH PROBLEMS

Despite the insights made possible by these methods, ABMs also have several important limitations and challenges that derive from the nature of ABM development and parameterization and warrant careful consideration when interpreting model results.

One of the foremost challenges in designing and implementing an ABM is the palpable tension between model simplicity and model realism. Modelers are repeatedly cautioned to follow the KISS principle (keep it simple, stupid), championed by Robert Axelrod (4), but they are also encouraged to take advantage of the complexity permitted in ABMs to capture critical elements of the system in well-defined populations in order to generate meaningful results for potential interventions and public health planning (53, 58). Finding a balance between the desire for simplified representations of reality and the need to include enough complex elements to provide new insights, then, becomes a true art (53), which is developed through trial and error and openness to adaptation in the face of mistakes. We must not be too wedded to the idea of our model that we fail to recognize when its complexity limits its meaningful interpretation, thereby defeating the purpose of modeling. Rather, we must take a sensible approach to building our models, gradually adding in complexity when warranted and working with diverse stakeholders to identify the essential elements needed for our model to be credible and useful (2, 25, 95). We can also consider including random effects in our model to capture other unspecified influences, as in Gorman and colleagues’ (43) and traditional statistical approaches, with the potential of further parsing out these effects in subsequent iterations of the model.

Compounding the uncertainties of model resolution are the challenges of model parameterization in the absence of empiric data. When empiric data are available, they often come from observational studies conducted in specific populations that may have different distributions of causal partners than the intended target population of the ABM (54, 103). As such, we may base our model specification on data that are subject to confounding as well as questionable transportability. There is a fundamental conundrum here: We want to use these models to gain insight into causal mechanisms and counterfactual contrasts, but we often rely on observational data from studies that were incapable of exploring causal mechanisms and counterfactual contrasts (26). Furthermore, we are often left with little to no empiric data on the very elements of the model that represent its greatest advantage over more traditional approaches: social network influences and the strength of interactions between units. These situations highlight the need for creativity in developing our models to reflect both quantitative and qualitative knowledge (85), for transparency about model assumptions and their implications (83), and for refined validation techniques to bolster confidence in our model results (53). Validation itself is challenging when empiric data are scarce because, ideally, data used for validation purposes should be independent of those used to build and calibrate the model.

In addition to the difficulties in defining the appropriate scope of the model and in parameterizing and validating it, simulating interventions and policies often proves challenging, though it is a key objective of this work. Many public health–related ABMs estimate population health outcomes assuming that interventions had a certain level of effect (for example, reduced unhealthy eating by 10% or 20%), but do not as of yet have sufficient data to simulate the steps of the intervention that would lead to such a reduction. Although these modeling exercises may be useful in making qualitative comparisons between interventions theorized or observed in randomized trials to have different magnitudes of effects, they do not advance the desired outcome of ABMs, which is to understand how and why different interventions are successful at improving population health (89).

Finally, as others have noted (2, 27), there are many logistical hurdles to successful implementation of ABMs in public health. Such hurdles include lack of training in these modeling techniques among public health students, researchers, and professionals (67, 71, 88), as well as the significant burden of time and computing resources needed to develop, run, and validate these models.

## FUTURE DIRECTIONS FOR AGENT-BASED MODELING IN PUBLIC HEALTH

Despite these limitations and challenges, ABMs remain a promising tool for informing public health research, practice, and policy. We now discuss future methodologic and substantive directions in agent-based modeling that we believe will move this field forward and address some of the challenges noted above.

### Improving the Methodology of ABMs in Public Health

Given the need for reproducibility as agent-based modeling becomes more widely used in public health, we echo others in calling for the widespread adoption of systematic protocols for calibration, verification, validation, and model reporting (2, 33, 95). Many ABM studies in public health follow the ODD (overview, design concepts, and details) protocol in describing the methods used (45, 46; for examples, see the supplementary materials of References 10 and 12), and we agree that this aids in understanding what was done in a particular simulation and how it could be replicated or extended. Given that the ODD protocol was developed by modelers in disciplines other than public health (primarily ecology) (46, 47), we note that the public health modeling community has much to learn about best practices in ABM methodology from other disciplines that have been involved in this work far longer. This is particularly true with respect to validation, which has been rather underdeveloped in public health ABM applications, contributing to skepticism and a perceived lack of robustness of these methods (100). Extensive reviews of ABM validation have been offered in other disciplines, including ecology and economics (36, 40, 64, 105). Besides increasing confidence in model results, validation techniques like uncertainty analysis and global sensitivity analysis have the potential to at least partially resolve the tension of model resolution discussed earlier, by elucidating whether particular parameters contribute to the explanatory power of the model or a simpler model would be just as informative (64). As others have noted (2, 75), ABMs in public health would benefit from the adoption of systematic protocols for ABM validation, including best practices identified in other disciplines such as observing model behavior when parameters are set to extreme values and behavioral rules are modified (20). Alternative approaches to empirical validation, including consideration of face validity and companion modeling [in which subject matter experts are involved in the modeling process and assessment of model credibility (20, 80)] may be particularly relevant for public health questions for which empiric data are lacking. To this end, academic-public partnerships may be critical in bringing together both the modeling and the substantive expertise necessary to develop a credible ABM for a particular public health problem (37). Creating accessible, user-friendly interfaces through which public health practitioners and policy makers could tailor ABM specifications to their particular settings would also promote further adoption of these methods, greater usefulness of the model results, and more opportunities for independent assessments of their credibility (5).

In addition to drawing on methodologic work from other disciplines, the field of public health offers its own unique methodological contributions to agent-based modeling. This includes efforts to formalize the role of these models in causal inference (75). In particular, explicit comparisons of ABMs with other causal modeling approaches like marginal structural models and the parametric g-formula would highlight the assumptions required by each method and the particular insights made possible by an agent-based modeling approach. Recently, Murray and colleagues (81) used a comparison of ABMs with g-formula strategies to estimate the causal effect of antiretroviral therapy on 12-month mortality among a simulated sample of persons with HIV. They demonstrated that ABMs result in bias when assumptions are not met regarding time-dependent confounding, mediation, and the transportability of causal estimates. Such limitations are not unique to ABMs, yet they highlight the methodological work that remains to be accomplished in promoting ABMs that yield insights for public health.

Many ABMs in public health utilize abstract representations of the physical environment, which may be perfectly appropriate if environmental influences are not of central importance to the model objectives. Following the lead of the MIDAS group and others (21, 101), however, public health modelers may want to consider the potential for additional insight through explicitly integrating in their models geographic information systems (GIS) data, which can be accommodated in most ABM software packages. The availability of big data from electronic medical records and mobile devices may also present opportunities for ABMs—for example, by including the use of cell phone data to simulate social network connections and interactions between individuals (58)—especially given the limited collection of network data in observational studies. One challenge in this area will be to continue weighing the value of increased model complexity against the need to derive meaningful inferences from the model output.

Model complexity in terms of multiscale interactions also presents unique challenges for agent-based modeling in public health. In many cases, these interactions are of central interest when modeling the system in which health arises: Are forces acting synergistically, antagonistically, or additively? However, data on these joint effects are scarce. Just as systems modeling may provide insight into the causal pathways leading to health outcomes (83), the agent-based modeling process itself presents an opportunity to systematically test alternative specifications of interaction effects in order to gain insight into plausible joint effects.

Finally, despite the advantages of ABMs in terms of highlighting the data that are needed to understand a particular public health system (1, 2), examples of data collection that have resulted from ABM exercises (which could then be used in future ABM development) are difficult to find. However, following the modeling cycle through its circular path (i.e., from model development to model implementation to data collection and back to model development) would present real opportunities to advance knowledge about public health problems. Similarly, our understanding of many public health problems would benefit from using complementary modeling approaches in an iterative fashion rather than relying too heavily on one statistical or systems science approach (54, 85). Many of the current ABM applications in public health end with tentative statements about their potential usefulness, with the implicit caveat that the model assumptions may be questioned and that empirical data grounding the model may be lacking. It is time we address these weaknesses through improved validation efforts, targeted data collection, and complementary modeling approaches in order to increase the payoffs from the modeling enterprise and to enable real conclusions about public health practice and policy.

### Broadening the Substantive Focus of ABMs in Public Health

In addition to methodological improvements, the substantive focus of current ABM applications also needs attention. We suggest that ABMs in public health would benefit from less of a focus on one particular health condition or behavior and more of a broader consideration of interrelated health conditions and behaviors. This would allow for the exploration of the net effects of given policies on population health (97) and lead to greater understanding of comorbidity and other adaptive relations between health behaviors and outcomes. We expect that formulating a life course approach within ABMs (e.g., evaluating the influence of experiences and interventions at different developmental stages on trajectories of disease) will be particularly fruitful in understanding the origin and perpetuation of health disparities (24). Finally, we would encourage researchers to include multiple types of risk factors in their models in order to identify linkages across scales; this may include genetics, biology, behavior, environment, and networks. Although empiric data may not be available to parameterize the joint effects of these multiple levels of influence and to model the interrelations between them, the process of modeling may provide insight into how these factors work together to produce disease. At the same time, an appropriate balance is needed in identifying the aspects of the system that are most relevant for a particular problem, rather than trying to model the system as a whole (85). With this balance in mind, expanding the application of ABMs in public health in these and other ways stands to increase our ability to understand and intervene to improve population health.
